# Crosstalk of angiogenesis-related subtypes, establishment of a prognostic signature and immune infiltration characteristics in colorectal adenocarcinoma

**DOI:** 10.3389/fimmu.2022.1049485

**Published:** 2022-11-24

**Authors:** Guoliang Cui, Jinhui Liu, Manli Wang, Kinyu Shon, Can Wang, Fei Wei, Zhiguang Sun

**Affiliations:** ^1^ Department of Gastroenterology, The Second Affiliated Hospital of Nanjing University of Chinese Medicine, Nanjing, Jiangsu, China; ^2^ Department of Gynecology, The First Affiliated Hospital of Nanjing Medical University, Nanjing, Jiangsu, China; ^3^ The First Clinical Medical College, Nanjing University of Chinese Medicine, Nanjing, Jiangsu, China; ^4^ Department of Colorectal Surgery, Affiliated Hospital of Nanjing University of Chinese Medicine, Jiangsu Province Hospital of Chinese Medicine, Nanjing, Jiangsu, China; ^5^ Department of Physiology, School of Medicine & Holistic Integrative Medicine, Nanjing University of Chinese Medicine, Nanjing, Jiangsu, China

**Keywords:** colorectal cancer, angiogenesis, prognosis, tumor microenviroment, risk score signature

## Abstract

**Background:**

Colorectal adenocarcinoma (COAD) is one of the most common malignancies and angiogenesis is vital to the development of cancer. Here, we explored the roles of angiogenesis-related genes (ARGs) that affect the prognosis of COAD and constructed risk models to assess patient prognosis, immune characteristics, and treatment outcomes.

**Methods:**

We comprehensively characterized the transcriptional and genetic modifications of 48 ARGs in COAD and evaluated the expression patterns. We identified two ARG subgroups using the consensus clustering algorithm. Based on the differentially expressed genes (DEGs) of two ARG subtypes, we calculated risk score, namely ARG_scores, and calssified COAD patients into different risk groups. To investigate the expression of ARG_score-related genes, qRT-PCR was performed. Subsequently, we mapped the nomogram to visually and accurately describe the value of the application of ARG_score. Finally, the correlation between ARG_score and clinical features, immune infiltration along with drug sensitivity were explored.

**Results:**

We identified two ARG related subgroups and there were great differences in overall survival (OS) and tumor microenvironment. Then, we created an ARG_score for predicting overall survival based on eight DEGs and confirmed its reliable predictive power in COAD patients, with higher ARG_score associated with worse prognosis. Furthermore, eight ARG_score-related genes expression was investigated by qRT-PCR. To make the ARG_score clinically feasible, we created a highly reliable nomogram. We also found a higher proportion of microsatellite instability-high (MSI-H) and higher tumor mutational burden (TMB) in the high-risk group. In addition, ARG_score was notably correlated with cancer stem cell indices and drug sensitivity.

**Conclusion:**

This scoring model has potential clinical application value in the prognosis, immune microenvironment and therapeutic drug sensitivity of COAD, which provides new insights for personalized treatment.

## Introduction

Colorectal cancer (CRC) is one of the most common malignant tumors. According to the latest global cancer statistics, CRC has the third highest incidence (10.0%) and the second highest mortality rate (9.4%) in the world (1). Moreover, the incidence of CRC is trending younger, with an annual increase of 2% in incidence and 1.3% in mortality among people under 50 years of age (2). It seriously endangers people’s lives and health, thus imposing a great socioeconomic burden worldwide.

With the continuous in-depth research on the pathogenesis and molecular mechanism of CRC, the treatment of this disease has made great progress. Surgical/polypectomy, chemotherapy and radiotherapy have benefited some CRC patients (3), but the tumor still has a high probability of local recurrence and metastasis. With the opening of the era of immunotherapy with PD-1 monoclonal antibody for metastasis colorectal cancer (mCRC), neoadjuvant therapy for CRC has seen a new dawn, but significant benefit has been observed only in selected patients (4). Therefore, there is a need to find accurate and reliable biomarkers to predict treatment response in the clinic for patients with different immune profiles.

The complex interactions between tumor cells and their microenvironment regulate the development and progression of cancer. The tumor microenvironment (TME) is composed of tumor cells, resident and recruited host cells (cancer-associated stromal cells and immune cells), as well as secreted substances of the corresponding cells (e.g. cytokines and chemokines) and non-cellular components of the extracellular matrix (ECM) (5), of which angiogenesis plays an important role. Angiogenesis is the physiological process of forming new blood vessels from pre-existing ones. This process provides oxygen and nutrients to the tumor, and excretes metabolic waste and carbon dioxide. During tumor progression, angiogenesis is always activated and contributes to tumor growth. In 2005, Prof. Jain first introduced the concept of “Normalization of tumor vasculature” (6). To date, some studies have also confirmed that the rational use of anti-angiogenic drugs, combining them with immune checkpoint inhibitors, can induce normalization of tumor blood vessels, improve the tumor microenvironment, and generate effective anti-tumor immunity (7). Several bioinformatics-based analyses have identified angiogenesis-related genes (ARGs) as potential prognostic biomarkers for several cancers (8–10).However, it is unclear whether ARGs can be used as potential prognostic markers for CRC.

CRC can be divided into colon and rectal cancer according to the primary tumor site. Colon adenocarcinoma (COAD) is the most common types of colon cancer among many pathologies. In this study, we collated samples from TCGA-COAD and GSE39582 cohort, identified different ARG subgroups, and developed a COAD prognostic scoring model, which can well differentiate the prognosis, immune characteristics, and treatment outcomes of patients with different risk scores. We further analyzed the sensitivity of chemotherapeutic agents of different risk scores. We hope that this study will contribute to the discovery of new diagnostic and prognostic biomarkers and new therapeutic targets for patients with COAD.

## Methods and materials

### Data collection

RNA expression, somatic mutation, copy number variation(CNV) datasets, and matching clinicopathological information for COAD were downloaded from the TCGA-COAD database (11), including 473 tumor datasets and 41 normal datasets. Clinical parameters and normalized gene expression data were acquired from GSE39582 in GEO database. Tumor samples from GSE39582 cohort and TCGA-COAD cohort were retained for further analysis. The batch effects between the TCGA and GEO datasets were removed using “ComBat” algorithm from the “sva” package (12). Based on a previous study, 48 ARGs were obtained from the MSigDB database (Hallmark Gene Set) (13).

### Consensus clustering analysis

To identify different angiogenesis patterns, clustering analysis was performed by k-means algorithm (14). The number and consistency of clusters were established by the consensus clustering algorithm in the “consuclusterplus” package (15). The process was repeated 1000 times to ensure the stability of these categories.

### Gene set variation analysis

To determine the biological functional differences of ARGs, we performed a gene set variation analysis (GSVA) based on “c2.cp.kegg.v6.2.symbols.gmts” in the MsigDB database (16).

### Assessment of tumor microenvironment

We used the ESTIMATE algorithm to estimate stromal scores and immune scores in COAD patients with the aim of assessing tumor purity (17). Next, the CIBERSORT algorithm was used to calculate the levels of 22 immune cell subtypes for each patient (18). The relative infiltration abundance of immune cells was calculated by single sample gene set enrichment analysis (ssGSEA) (19). Then, we evaluated the expression of two immune checkpoints, PD-L1 and CTLA-4, in two clusters.

### DEGs identification and functional enrichment analysis

We used “limma” package to identify differentially expressed genes (DEGs) in different angiogenesis subgroups, and its standard is | log2-fold change (FC) | ≥ 2, P value < 0.05. Then used the “clusterprofiler” package for GO and KEGG analysis (20).

### Construction of the angiogenesis-related prognostic ARG_score

In order to quantitatively evaluate angiogenesis in each COAD patient, we constructed a scoring model called ARG_score (risk score). All COAD patients were randomly separated into training cohort (n=594), testing cohort (n=396) and entire cohort (n=990). There were no significant differences in clinicopathological factors among the three cohorts (**Supplementary Table 1**). The expression data of DEGs from different angiogenesis clusters were normalized, and the intersecting genes were selected. Differential assessment revealed 1587 DEGs between the two angiogenesis clusters. Next, we performed univariate Cox regression (unicox) analysis on the DEGs in the training cohort. 466 prognosis related genes were reserved for further analysis, and then the 466 survival related genes were analyzed by lasso and multivariate Cox (multicox), and finally 8 candidate genes were included in this angiogenesis-related signature. ARG_scores (risk socre) were calculated using the following method: ARG_ Score = gene expression (1) × Corresponding coefficient (1) + gene expression (2) × Corresponding coefficient (2) + gene expression [n] × Corresponding coefficient [n]. The patients were categorized into high-risk and low-risk groups based on the median score. Survival analysis were performed using “survminer” package and the receiver operating characteristic (ROC) curve was used to reveal the predictive effect of this model. Moreover, we also performed same analysis on testing and entire cohort.

### Cell culture and qRT-PCR

Caco-2, HT-29, HCT-116, the human colorectal cancer cell lines, were obtained from the China Center for Type Culture Collection (CCTCC, Wuhan, China) and cultured in McCoy’s 5A, RPMI-1640, high-glucose DMEM medium (Gibco, Shanghai, China) respectively. FHC, the normal colon epithelial cell line, was purchased from the Cell Bank of Type Culture Collection of the Chinese Academy of Sciences (Shanghai, China) and cultured in RPMI-1640 (Gibco, Shanghai, China). The medium were supplemented with 10% fetal bovine serum (FBS, Gibco, Shanghai, China) and 1% antibiotics. All cells were incubated at 37°C with 5% CO_2_. Total RNA was isolated by TRIZOL reagent and cDNA was synthesized (Vazyme, China). β-actin was selected as an internal reference. The 2^−ΔΔCT^ method was used to estimate the relative expression of target genes. Primer sequences are listed in **Supplementary Table 2**.

### Clinical significance and classification analysis of ARG_score

We performed uniCox and multiCox analyses on all cohorts to determine whether ARG_score could be used as an independent prognostic factor. Afterwards, to explore whether the predictive function of ARG_score was reliable in different subgroups of clinical variables, a classification analysis was performed. Moreover, the levels of immune cells and immune checkpoints (ICPs) in different risk score subgroups were compared. In addition, we investigated the correlation between ARG_score and tumor mutational burden (TMB) score, microsatellite instability (MSI) score and cancer stem cell (CSC) score. One-class logistic regression (OCLR) machine-learning algorithm was used to quantify the stemness of tumor samples by calculating cancer stem cell indices (21).

### Creation and validation of nomogram

To predict the 1-year, 3-year and 5-year OS of each COAD patient, we integrated ARG _score and other clinicopathological features to create a nomogram with the “rms” package (22). And then, calibration curve analysis was used to assess the predictive power of the model (23).

### Mutation and drug sensitivity analysis

We used the “maftools” package to create a mutation annotation format (MAF) in the TCGA database to understand the genetic variants in COAD patients in different risk groups (24). In addition, to investigate the sensitivity of chemotherapeutic drugs in different risk groups, we calculated the semi-inhibitory concentration (IC50) values of common drugs using the “pRophetic” package (25). We also analyzed the correlation of eight ARGs with the sensitivity of commonly used chemotherapeutic agents.

### Statistical analysis

Data processing, analysis and presentation were carried out with R software (version 4.1.2). The prognosis survival curve was drawn by Kaplan Meier plotter. Spearman analysis was used for correlation analysis. P <0.05 means the results are statistically significant.

## Results

### Expression and mutation of angiogenesis-related genes in COAD

**Figure 1:**
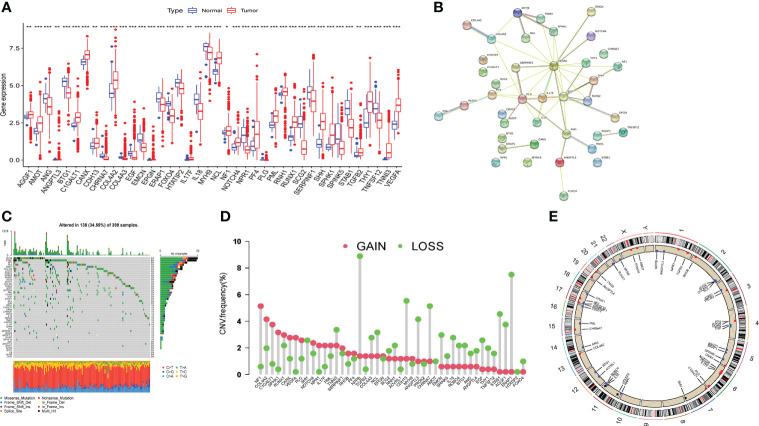
Expression and mutation of angiogenesis related genes in TCGA-COAD cohort. **(A)** Differential expression of angiogenesis related genes (ARGs) between tumor tissue and normal tissue. **(B)** The protein-protein interaction (PPI) network of the differentially expressed genes. **(C)** The incidence of somatic mutations of ARGs in COAD patients. **(D)** The CNV frequency of ARGs in TCGA cohort. **(E)** The locus of CNV alterations of 48 ARGs on 23 chromosomes. Adjusted p-values were shown as *P<0.05, **P<0.01, ***P<0.001.

To find out whether genetic variants in ARGs are associated with COAD, we determined the mutation landscape of ARGs. From MSigDB database, 48 angiogenesis-related genes (ARGs) were included in this study according to the previous study (13), of which a total of 40 differentially expressed genes (DEGs) were identified between normal and tumor tissues (**Figure 1A**). The protein-protein interaction (PPI) network established by the STRING website showed the interactions of these DEGs (**Figure 1B**). We then recognized the incidence of somatic mutations in these ARGs, and the result revealed that among the 399 samples, 138 samples carried mutant ARGs, with a mutation rate of 34.59%. Meanwhile, MYH9 and STAB1 had the highest mutation rate (**Figure 1C**). In addition, we confirmed the prevalence of CNV alterations in ARGs in COAD patients (**Figure 1D**), and CNVs seem associated with higher expression of ARGs in tumor tissues, such as NF1, COL4A2, C1GALT1, SPHK1, RUNX1, implying a potential regulatory role of CNVs on the expression of ARGs (**Figure 1D**). **Figure 1E** showed the CNVs locus of 48 ARGs on 23 chromosomes. The above study indicated that the expression and mutation of ARGs differed greatly between normal and COAD samples, suggesting that ARGs may played an important role in the COAD.

### Formation of angiogenesis-related genes clusters

**Figure 2:**
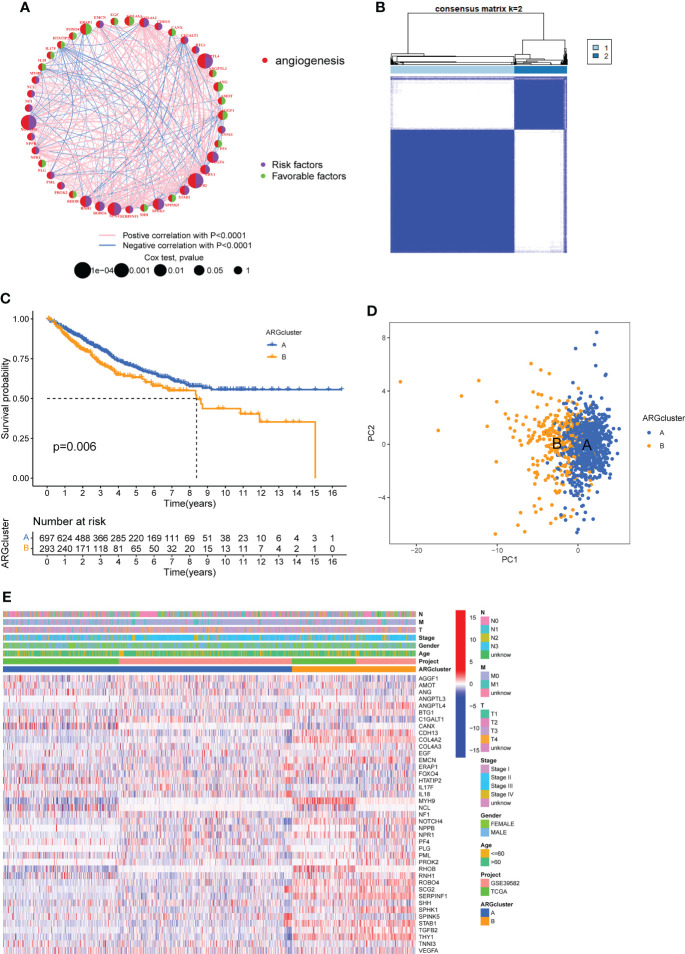
Formation of angiogenesis-related genes clusters (ARGclusters). **(A)** The network showing the correlation of ARGs in COAD. **(B)** All samples from TCGA-COAD cohort and GSE39582 cohort were divided into 2 clusters using consensus clustering algorithm (k = 2). **(C)** Kaplan-Meier curves show the different overall survival (OS) between two ARGclusters. **(D)** Principal component analysis (PCA) showed significant differences between the two ARGclusters. **(E)** Heatmap showed the differences between two clusters in clinical information and ARGs expression.

To explore the survival significance of ARGs, we integrated samples from TCGA-COAD and GSE39582 cohort, and investigated the expression levels of ARGs in relation to overall survival (OS) by using Kaplan-Meier analysis, the results showed that 31 ARGs were related with OS and higher expression of most genes (22/31) implied a worse prognosis in COAD patients (**Supplementary Figure 1**). The interactions and risk/favorable factors of ARGs in COAD were exhibited **Figure 2A**, showing the complex crosstalk of these prognosis-related ARGs. To determine the subtypes of COAD, we used the consensus clustering algorithm to classify the samples according to the expression of ARGs and divided the integrated cohort into two clusters, namely ARGcluster (ARGcluster A and ARGcluster B) (**Figure 2B**), the survival analysis revealed that ARGcluster A had a better survival expectation (**Figure 2C**). Principal component analysis (PCA) confirmed a significant difference in the distribution of the two ARGclusters (**Figure 2D**). Furthermore, we compared the gene expression and clinical information of the two ARGclusters, found that the expression of ARGs differed significantly, with most ARGs being more highly expressed in ARGcluster B (**Figure 2E**). The above results showed that the two ARGclusters had significant differences in OS and ARGs expression, with clusterB having higher ARGs expression and poorer survival expectations.

### Difference of biological features and tumor immune infiltration between two ARG clusters

**Figure 3:**
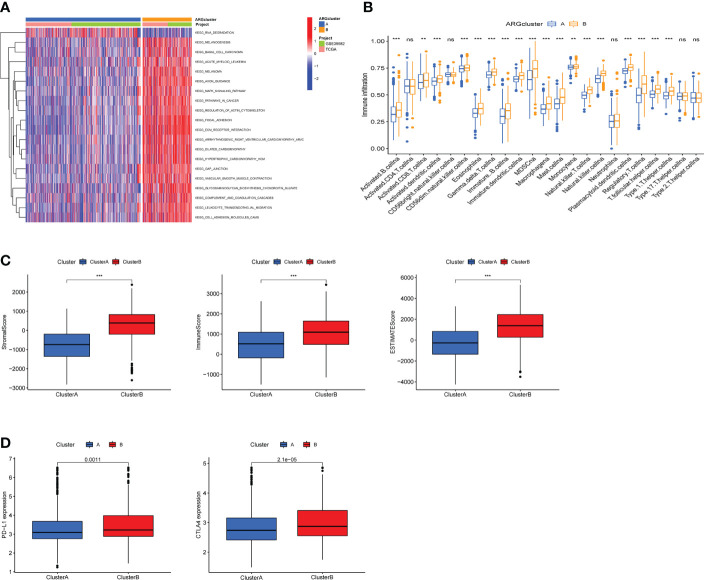
Analysis of biological features and tumor immune infiltration in two ARGclusters. **(A)** KEGG-related GSVA analysis showing the biological pathways of two ARGclusters. **(B)** Infiltration of 23 types of immune cells in two ARGclusters. **(C)** Differences of stromal score, immune score and ESTIMATE score between the two ARGclusters. **(D)** Expression levels of immune checkpoints PD-L1 and CTLA-4 in the two ARGclusters. Adjusted p-values were shown as ns, nosignificant, **P<0.01, ***P<0.001.

In order to understand the differences in the biological functions of the two ARGclusters, KEGG-related GSVA analysis was performed. The results indicated that cell proliferation and differentiation-related (MAPK signaling pathways) and metastasis-related (focal adhesion, ECM receptor interaction) were more abundant in ARGcluster B (**Figure 3A**). The abundance of immune cells affects the tumor microenvironment and regulates tumors development, therefore we compared the abundance of immune cell subpopulations in two ARGclusters with ssGSEA. The abundance of 18 immune cell subpopulations were statistically different in the two ARGclusters, and all are more highly expressed in ARGcluster B (**Figure 3B**). Furthermore, we performed the ESTIMATE algorithm to infer differences in stromal score and immune score between the two clusters, and it turned out that ARGcluster B was significantly abundant in immune cells and stromal cells (**Figure 3C**), which means cluster B has relatively lower tumor purity. Besides, the expression of immune checkpoint genes PD-L1 and CTLA4 in ARGcluster B were also notably higher than that in ARGcluster A (**Figure 3D**).These results further comfirmed the differences between two ARGcluster, such as biological characteristics and tumor microenvironment, higher infiltration of immune cells and higher ESTIMATE score were found in clusterB.

### Construction gene clusters based on angiogenesis-related DEGs

**Figure 4:**
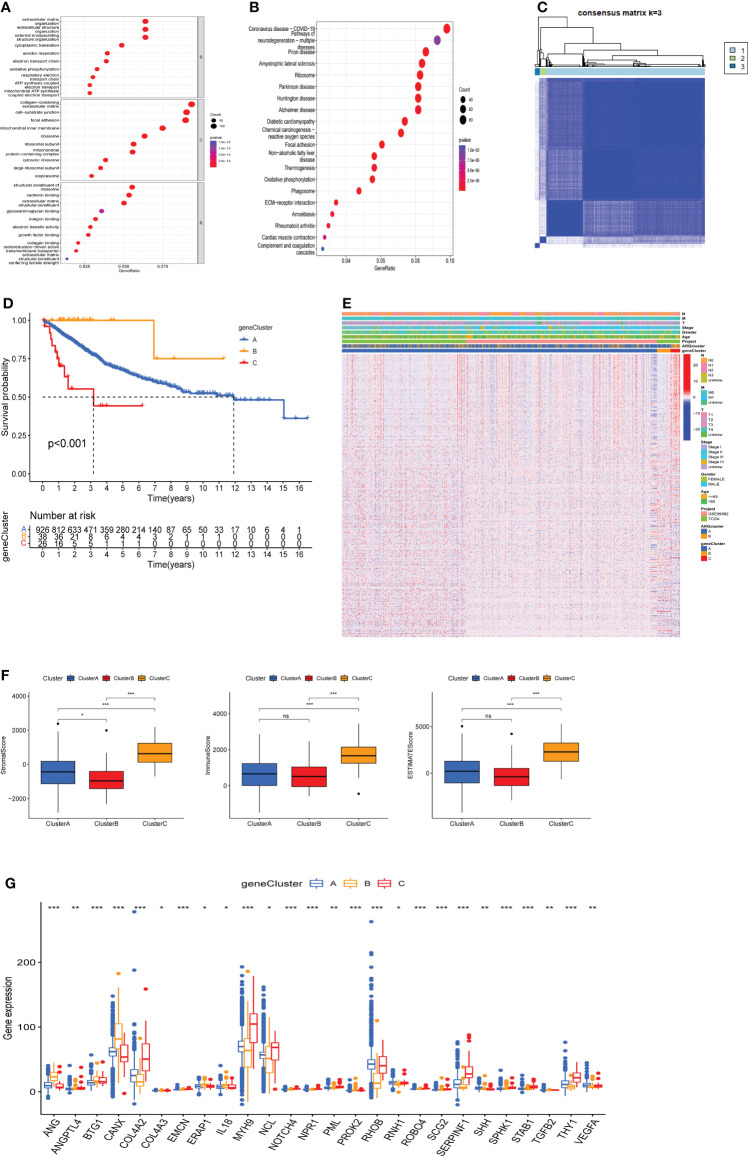
Construction of gene clusters based on the differentially expressed genes (DEGs) and analysis of prognosis, pathological features and tumor immune microenvironment (TME) in gene clusters. **(A)** Gene Ontology (GO) enrichment analysis of DEGs between two ARGclusters. **(B)** Kyoto Encyclopedia of Genes and Genomes (KEGG) enrichment analysis of DEGs between two ARGclusters. **(C)** The consensus matrixes for TCGA-COAD cohorts based on the DEGs among the 2 ARG clusters(k=3). **(D)** Kaplan-Meier analysis showing the different OS of the three gene clusters. **(E)** Heatmap shows the different clinicopathological features of the three gene clusters. **(F)** Differences of stromal score, immune score and ESTIMATE score between the 3 gene clusters. **(G)** Expressions level of ARGs in three gene clusters. Adjusted p-values were shown as ns, no significant, * *P* < 0.05, ** *P* < 0.01, *** *P* < 0.001.

To further investigate the potential biological behavior of each angiogenesis subgroup, we identified 1587 DEGs between two ARGclusters using the “limma” package (**Supplementary Figure 2**), and performed functional enrichment analysis on these DEGs. GO and KEGG enrichment analysis indicated that these DEGs were mainly enriched in tumor metastasis-related pathways (**Figures 4A, B**). To determine the prognostic value of these DEGs, uniCox analysis was performed on 1587 DEGs and 466 DEGs associated with prognosis were screened out with a criterion of *p* < 0.05. Based on prognosis-related DEGs, patients from TCGA-COAD cohort and GSE39582 cohort were divided into three clusters (namely, gene cluster A, B, and C) using the consensus clustering algorithm (**Figure 4C**). Survival analysis showed that gene cluster B had the best prognosis, while cluster C had the opposite (**Figure 4D**). Heatmap reflected the expression level of prognosis-related DEGs and the difference of clinicopathological factors in two ARGclusters and three gene clusters (**Figure 4E**). We also used the estimate algorithm to determine the differences in stromal score, immune score and ESTIMATE socre between three gene clusters. The immune cells and stromal cells in cluster C were significantly more than those in the other two gene clusters, while the stromal cells in group B were the least (**Figure 4F**). In addition, the expressions of prognosis-related AGRs in the three gene clusters were investigated, 26 of the 31 prognosis-related AGRs were differentially expressed. (**Figure 4G**). The above results reflect the differences in survival expectancy, clinical characteristics, and ESTIMATE scores among the three gene clusters, which also proved that DEG between two ARGclusters well distinguished the prognosis and immune microenvironment of COAD patients.

### Construction and validation of the prognostic ARG_score model

**Figure 5:**
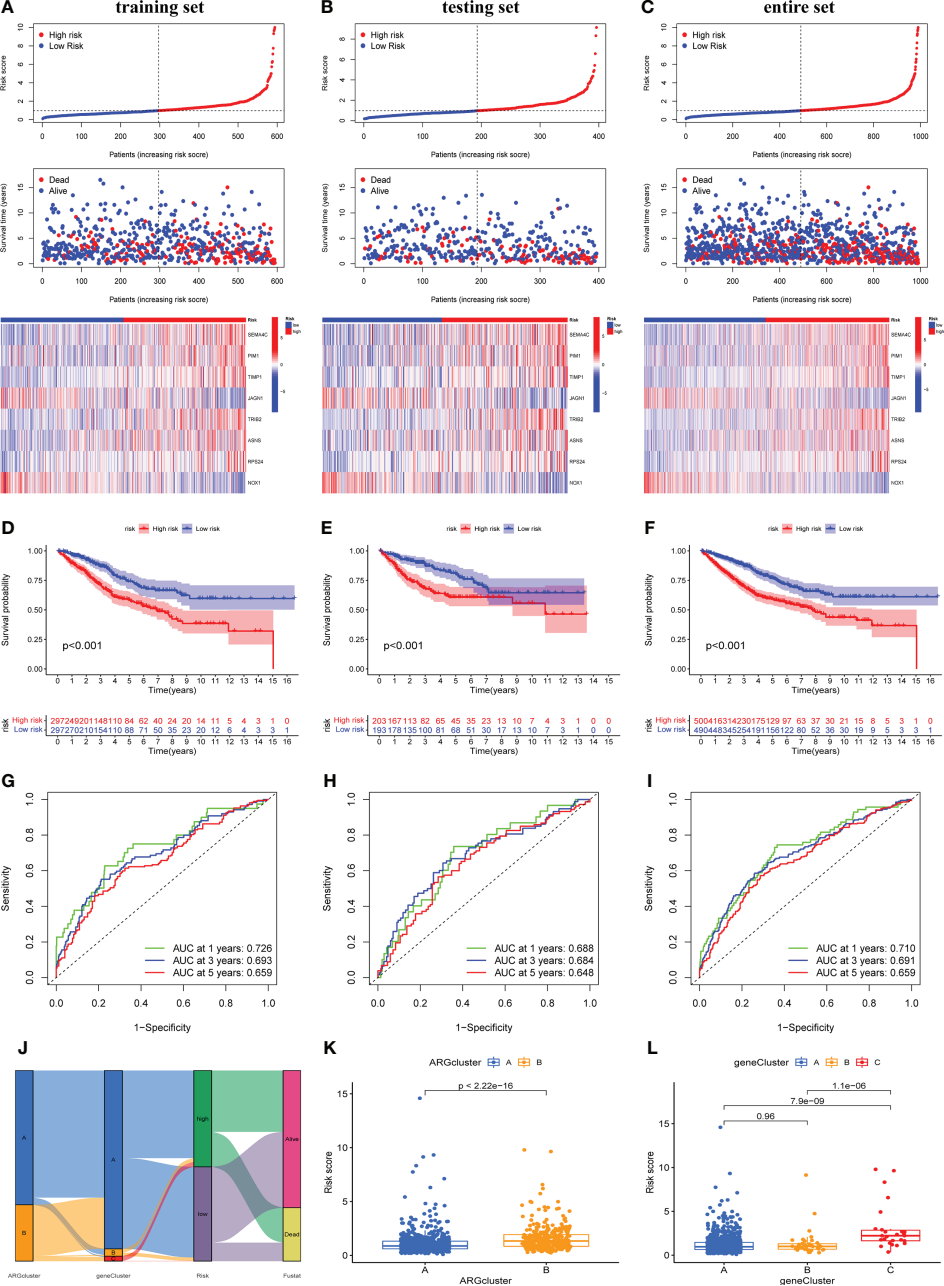
Construction and validation of the prognostic ARG score model. Significant differences in survival time and expression of 8 prognosis-related genes between high-risk and low-risk groups in training cohort **(A)**, testing cohort **(B)** and entire cohort **(C)**. Survival analysis of the overall survival (OS) for high-risk and low-risk patients in training cohort **(D)**, testing cohort **(E)** and entire cohort **(F)**. The ROC curves for 1-,3-,5-years survival of COAD patients in training cohort **(G)**, testing cohort **(H)** and entire cohort **(I)**. **(J)** Alluvial plot shows the distribution of patients in two ARGclusters, three gene clusters, two risk groups and their survival status. The differences in risk score of two ARGclusters **(K)** and three gene clusters **(L)**.

To predict the outcome of each patient with COAD, we created a scoring model based on prognosis-related DEGs between two ARGclusters, called ARG score (risk score). We randomly divided all the COAD patients into training cohort and testing cohort, and performed LASSO and multiCox analysis on 466 prognosis-related DEGs in the training cohort to build the prognostic model (**Supplementary Figure 3**). The LASSO Cox regression model was used to narrow the most robust ARGs for prognosis and ten-fold cross-validation was applied to overcome the over-fitting. To generate a prognostic signature model (risk score), multivariate Cox regression analysis was applied to evaluate the connection between ARGs and OS in the training set. At last, we constructed a risk signature in the light of 8 ARGs. Eight ARGs were finally included, namely SEMA4C, PIM1, TIMP1, JAGN1, TRIB2, ASNS, RPS24, NOX1. The formula for calculating the ARG score was: ARG score/Risk score = [expression of SEMA4C*0.0765] + [expression of PIM1*0.0304] + [expression of TIMP1 * 0.0035] + [expression of JAGN1 * (-0.03625)] + [expression of TRIB2 * 0.0332] + [expression of ASNS * 0.0546] + [expression of RPS24 * 0.0049] + [expression of NOX1 * (-0.0076)]. According to the median value of ARG_score, COAD patients were divided into high-risk and low-risk groups. **Figure 5A** showed the distribution, survival status of patients and the expression of eight genes in different risk groups in training cohort. With the increase of risk score, the survival time of patients decreased and the mortality increased. In the high-risk group, the expression of SEMA4C, PIM1, TIMP1, TRIB2, ASNS, RPS24 was higher, while the expression of JAGN1 and NOX1 was lower (**Figure 5A**). Furthermore, we performed survival analysis on the training cohort, which indicates that patients with higher risk scores had lower OS (**Figure 5D**). ROC curve shows that the prediction model had good sensitivity and specificity, the AUC values of 1-, 3-,5-years were 0.726, 0.693 and 0.659 respectively (**Figure 5G**). We conducted the same analysis on testing cohort and entire cohort, and similar results confirmed the accuracy of the risk score model (**Figures 5B, C, E, F, H** **, I**). To visually reflect the distribution of patients in two ARGclusters, three gene clusters, two risk score groups and their survival status, we constructed an alluvial plot (**Figure 5J**). Moreover, we compared the differences in risk scores of different subgroups. For ARGclusters, ARGcluster B had a higher risk score, and as for gene clusters, gene cluster C had the highest risk score (**Figures 5K, L**).

In addition, we verified the expression of 8 ARG score-related genes in CRC cell lines including Caco-2, HT-29, and HCT-116 by qRT-PCR (**Supplementary Figure 4**). Compared with normal colon epithelial cells, SEMA4C and ASNS expression were significantly increased in three CRC cell lines. PIM1, JAGN1 and RPS24 expression were significantly increased in Caco-2 and HT-29, but not in HCT-116. TRIB2 expression was significantly increased in HT-29 and HCT-116, but not in Caco-2. In this section, we developed the ARG-related score model, confirmed its good predictive value and verified the expression level of eight candidate ARGs in CRC cell lines

### Correlation between clinical pathological factors and risk score

We discussed the relationship between several clinicopathological factors (survival status, age, gender, stage) and risk score. It turned out that the mortality of high-risk group was significantly higher, and the proportion of advanced stage in high-risk group was higher (**Supplementary Figures 5A–D**). In addition, survival analysis was used to analyze the prognosis of patients in high-risk or low-risk groups with different pathological feature. The results show that the OS of COAD patients in the high-risk group was markedly lower than that in the low-risk group, regardless of age, gender or tumor stage. (**Supplementary Figures 5E–G**). The tumor primary sites in the cecum, ascending colon, and hepatic flexure are right-sided CRC (RCRC), while the tumor primary sites in splenic flexure, descending colon, sigmoid colon, and rectosigmoid junction are left-sided CRC (LCRC) (26). Compared with low-risk group, the proportion of RCRC patients in the high-risk group was higher, and the risk score of patients with RCRC was significantly higher than that of LCRC patients (**Supplementary Figure 6**).

### Construction and validation of a nomogram

**Figure 6:**
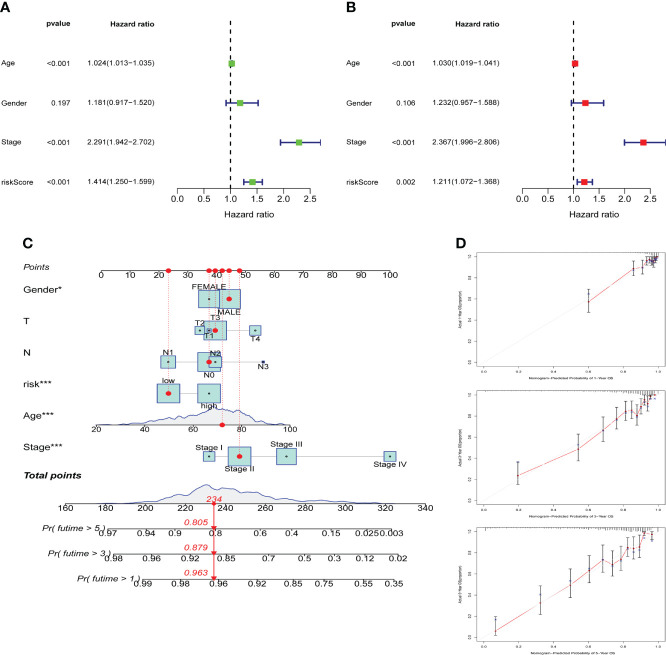
Construction and validation of a nomogram. Univariate cox regression **(A)** and multivariate cox regression **(B)** analysis of risk scores and clinicopathological factors. **(C)** Nomogram construction for predicting the 1-,3-,5-years OS of COAD patients. **(D)** Calibration curve analysis for predicting patients’ survival at 1-,3-,5-year, the grey line represents the ideal performance, and the actual performance of the signature is represented by the red lines.

We further explored whether individual pathological factors had independent prognostic value, and both uniCOX and multiCOX analysis show that age, stage, and risk score have independent prognostic value in the entire cohort (**Figures 6A, B**). Based on the correlation between the above clinicopathological features and ARG_scores, we created a nomogram for predicting 1-, 3-, and 5-year survival in COAD patients (**Figure 6C**). Calibration curve show that the nomogram was able to make accurate predictions (**Figure 6D**).

### ARG_score was correlated with tumor microenvironment and immune infiltration

**Figure 7:**
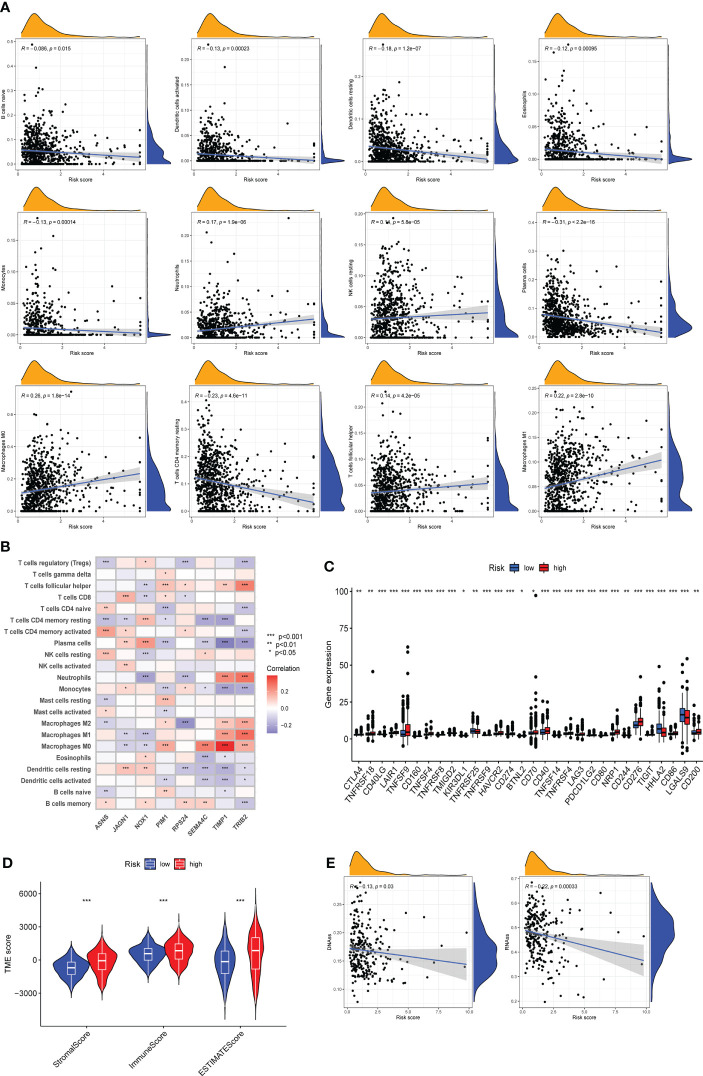
Assessment of tumor microenvironment (TME) and immune infiltration between different risk groups. **(A)** CIBERSORT algorithm reveals the correlation of risk score and immune cell subtypes. **(B)** Correlation between 8 candidate genes and immune cell abundance. **(C)** Expression levels of immune checkpoints in high-risk and low-risk groups. **(D)** Differences of Stromal score, Immune score and ESTIMATE score between the two risk groups. **(E)** Correlation of two cancer stemness cell indices (RNAss and DNAss) with risk score. Adjusted p-values were shown as * *P* < 0.05, ** *P* < 0.01, *** *P* < 0.001.

A large number of immune cells tend to accumulate in and around tumors, and these immune cells have complex interactions and regulation with tumor cells (27). Using the CIBERSORT algorithm to assess the relationship between the degree of infiltration of immune cell subtypes and the risk score, we found that the immune cell subtypes were positively associated with the ARG_score, including neutrophils, resting NK cells, Macrophages M0, T follicular helper cells, and Macrophages M1, while naive B cells, activated dendritic cells, resting dendritic cells, eosinophils, monocytes, plasma cells, resting memory CD4+ T cells are inversely correlated (**Figure 7A**). The correlation heatmap between the 8 candidate DEGs and immune cell abundance showed that most immune cells had an outstanding correlation with these 8 genes (**Figure 7B**). Moreover, the results based on ssGSEA confirmed that there were significant differences in some immune cells and immune function between high-risk and low-risk groups (**Supplementary Figure 7**). We further evaluated the expression of 30 ICPs in different risk groups and most ICPs were more expressed in the high-risk group (**Figure 7C**). In addition, we also evaluated the TME scores in both two risk groups, not surprisingly, stromal score, immune score, and ESTIMATE score were higher in the high-risk group than in the low-risk group (**Figure 7D**). Tumor stemness index is an index to assess the similarity between tumor cells and stem cells, which is related to active biological processes in tumor cells, such as cancer recurrence, tumor proliferation and drug resistance (28). Therefore, we evaluated the correlation between DNA stemness score (DNAss) along with RNA stemness score (RNAss) and risk score. The results showed that the risk score was significantly negatively correlated with DNAss and RNAss, implying that COAD cells suggestive of lower ARG scores had more prominent stem cell characteristics and lower levels of cell differentiation (**Figure 7E**).

### Association between ARG_score and Tumor mutation burden along with microsatellite instability

**Figure 8:**
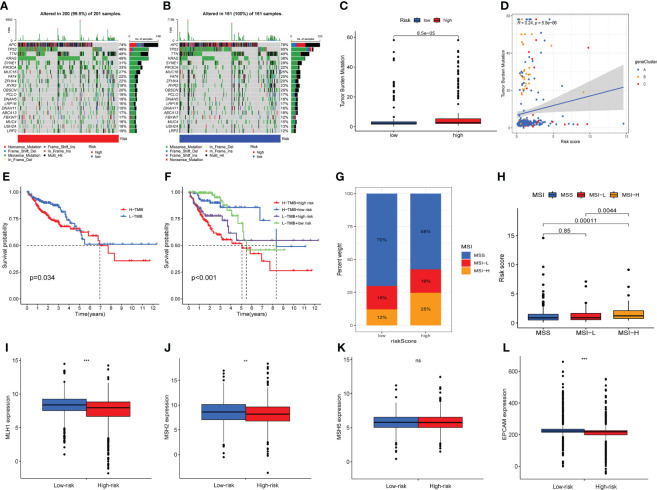
Association between ARG score and tumor mutation burden (TMB) along with microsatellite instability (MSI). The waterfall plot of 20 genes with the highest mutation rates in high-risk **(A)** and low-risk groups **(B)**. **(C)** Differences in tumor mutational burden between high-risk and low-risk groups. **(D)** Correlation between tumor mutational burden and risk score. **(E)** Survival analysis shows the OS differences between two TMB subgroups. **(E)** Survival analysis shows OS differences stratified by TMB and risk score. **(F)** The proportion of MSS, MSI-L and MSI-H in different risk groups. **(F)** Differences in risk score between MSS, MSI-L and MSI-H subgroups. **(I–L)** Expression levels of four mismatch repair related genes in different risk groups. Adjusted p-values were shown as ns, no significant, ** *P* < 0.01, *** *P* < 0.001.

We explored the differences in somatic mutations between two risk groups based on TCGA-COAD dataset. Top 20 genes in terms of mutation rate were exhibited in the waterfall plot (**Figures 8A, B**). The waterfall plot displayed that genes of top 3 mutation rate were APC, TP53 and TTN in two risk groups. Moreover, the mutation frequency of APC, TP53 and TTN was higher in the low-risk group. TMB could help predict patient response to immunotherapy, so we next analyzed the difference of TMB between the two risk groups, and found that the TMB level was higher in the high-risk group than in the low-risk group (**Figure 8C**). Furthermore, there was a positive correlation between ARG_score and TMB (**Figure 8D**). A prognosis analysis was implemented on TMB of two risk groups and turned out that patients with lower TMB have a better OS (**Figure 8E**). In addition, we analyzed the OS taking together TMB with ARG_score, indicating that high-TMB along with high-ARG_score presents the worst OS among the four groups (**Figure 8F**). The microsatellite instability (MSI) accompanied by defective DNA mismatch repair was an important prognostic marker for tumors in clinical practice, so we did a series of analyses related to MSI for different risk groups. As is shown in **Figure 8G**, the proportion of MSI-high (MSI-H) was higher in the high-risk group than in the low-risk group, while the proportion of MSS (microsatellite stability) was lower than in the low-risk group. The risk score in the MSI-H subtype was significantly higher than that in MSS and MSI-low (MSI-L) subtypes (**Figure 8H**). The expression of the four mismatch repair (MMR) related genes, except MSH6, the other three (MLH1, MSH2, and EPCAM) were significantly higher in the low-risk group than in the high-risk group (**Figures 8** **I–L**). MMR or MSI status is still the most important molecular marker to predict the efficacy of immunotherapy for colorectal cancer (29). Our analysis results were helpful to predict the effect of immunotherapy, and COAD patients with higher risk scores may have better effect on immunotherapy.

### Drug sensitivity analysis

**Figure 9:**
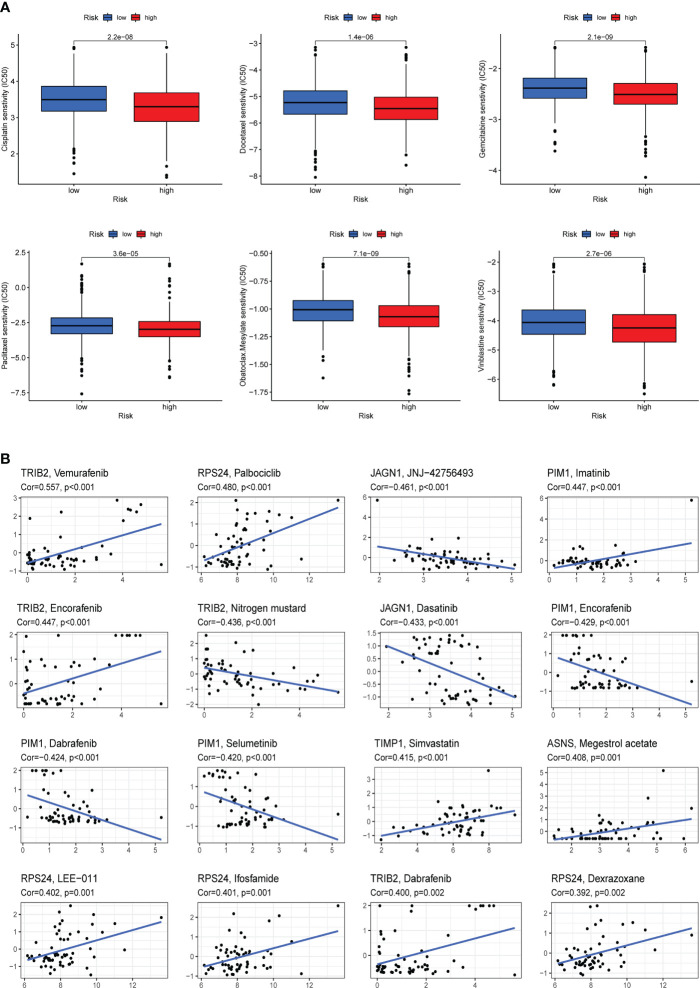
Drug sensitivity analysis. **(A)** Cisplatin, Docetaxel, Gemcitabine, Paclitaxel, Obatoclax.mecylate, and Vinblastine were observed to have lower IC50 values in the high-risk group, that is, the high-risk group was more sensitive to these drugs. **(B)** Analysis of the correlation between the expression of eight candidate genes included in the risk score model and chemotherapy drug sensitivity.

To explore the differences in sensitivity to chemotherapeutic drugs in two risk groups, we evaluated the IC50 values of drugs, and the results showed that patients with higher ARG_score were more sensitive to Cisplatin, Docetaxel, Gemcitabine, Paclitaxel, Obatoclax.mecylate, and Vinblastine (**Figure 9A**). Moreover, we analyzed the correlation between the expression of eight candidate genes included in the risk score model and the sensitivity of chemotherapeutic drugs. For instance, our results revealed a positive correlation between the expression of TRIB2 and the sensitivity of Vemurafenib, Encorafenib, and Dabrafenib, while a negative correlation with the sensitivity of Nitrogen mustard (**Figure 9B**). The results suggested that ARGs were correlated with drug sensitivity and lower ARG_score in this model suggests better treatment outcomes for patients with COAD.

## Discussion

In recent years, more and more immunotherapeutic methods have been applied to the treatment of tumors as the research on immunotherapy has intensified. However, satisfactory efficacy have been observed only in CRC patients with defective mismatch repair (dMMR) or high microsatellite instability (MSI⁃H), while pMMR-MSI-L CRC patients are insensitive to immunotherapy (4, 30).Therefore, it is of great importance to find new biomarkers at the molecular level to predict the prognosis of CRC patients, so as to guide clinical treatment, improve patient prognosis and prolong their survival time.

In this study, we developed and validated a risk score model capable of predicting survival in COAD patients based on the angiogenesis-related genes (ARGs). The model can robustly predict the clinical prognosis of patients, which is related to tumor microenvironment (TME) and immune characteristics. In addition, we also found that the ARG_score model can distinguish the sensitivity of patients with different risks to treatment, which indicates that the model has application value in clinical efficacy.

First, we identified ARGs mutations and expression in the TCGA-COAD cohort. Most of them are upregulated in COAD patients and associated with worse prognosis, suggesting a potential role of ARGs in COAD. Then, we divided COAD patients from TCGA-COAD cohort and GSE39582 cohort into two angiogenesis-related cluster (ARGcluster A and B) using the consensus clustering algorithm. There are significant differences in ARGs expression, OS and TME between the two ARGclusters. In addition to malignant tumor cells, tumor tissue includes various types of cells (immune cells, fibroblasts, endothelial cells, etc.), intercellular stroma, and extracellular factors (cytokines, chemokines, and growth factors) (31). These components and their complex interactions form the tumor-associated microenvironment. It is well known that the immune system has both pro-cancer and anti-cancer effects. There is a complex biological process between immune cells and malignant tumor cells in the tumor stroma with significant prognostic relevance (32). In colorectal cancer, the distribution, tissue localization, and cell type of different types of immune cells are significantly associated with tumor progression. In this study, the immune infiltration level of 23 human immune cell subpopulations in 2 clusters was assessed using ssGSEA, of which 18 immune cells were all more infiltrated in cluster B.

To quantify the angiogenesis subgroups, a scoring model, namely ARG_score, was constructed using LASSO and multivariate cox regression analysis. Among the two ARG_clusters and three gene clusters, ARG_cluster B and gene cluster A with the highest risk score have the worst prognosis, while ARG_cluster A and gene cluster B with the lowest risk score have the best prognosis. This indicates that the higher the ARG_score, the worse the prognosis. Our results show remarkable differences in genomic alterations between the low-risk and high-risk groups, with the expression of SEMA4C, PIM1, TIMP1, TRIB2, ASNS, and RPS24 being higher in the high-risk group, while the expression of JAGN1 and NOX1 was higher in the low-risk group. Previous studies have had similar findings. Semaphorins (SEMAs) are membrane-bound or soluble proteins involved in organ development and cancer progression, and among the SEMAs differentially expressed in colon cancer tissues, patients with tumors with higher SEMA4C (Semaphorins-4C) expression have lower survival rates (33). PIM1 expression is positively associated with CRC progression, and it was found to promote CRC growth and metastasis (34). Overexpression of TRIB2 accelerates cancer cell growth, cell cycle progression, and is associated with poor prognosis of CRC patients (35). High expression of asparagine synthetase (ASNS) is associated with poorer survival in women with right-sided colon cancer (RCC) (36). RPS24 is a gene that significantly promotes CRC cell proliferation (37), and knockdown of RPS24 can inhibit colorectal cancer cell migration and proliferation *in vitro* (38).

Both univariate and multivariate cox regression analysis showed that ARG_score was an independent predictor of survival outcome in COAD patients. The ROC validated its predictive robustness for 1-, 3- and 5-year OS. Thus, ARG_score may have reliable predictive power for COAD patient prognosis. We also analyzed the correlation between ARG score as well as ARG-related prognostic genes and immune cell infiltration. The results suggested that both ARG_score and candidate genes were strongly correlated with immune cells. Infiltration of some immune cells, such as dendritic cells (DCs) and CD4+ memory T cells, decreased as the risk score increased, while others, such as macrophages M0 and M1, did the opposite. These cells play complex roles in tumor immunity. For example, DCs are able to mediate cross-priming of tumor-specific T cells, which is essential for initiating and maintaining anti-tumor immunity. In tumors, the presence of DCs often induces T-cell response and mitigates cancer progression (39). In TME, macrophages, also known as tumor associated macrophages (TAMs), are one of the most abundant immune cells, which play an indispensable role in promoting tumor immune escape and inhibiting the immune function (40). Antitumor M1 TAMs and tumor-promoting M2 TAMs coexist in the TME (41). The interaction between M1/M2 TAMs directly affects the progress of CRC tumors and clinical treatment strategies (42, 43). One of the mechanisms of tumor immune escape is the metabolic reprogramming of TAMs, which prevents the increased inflammatory response mediated by M1 TAMs from killing tumor cells (44). Regulating the transformation of M2 TAMs into M1 TAMs has become a new direction for targeted treatment of tumor diseases (41, 45). In this study, the proportion of TAMs was significantly higher in ARGcluster B and high-risk group. Moreover, positive association was comfirmed between ARG_score and M1 TAMs, while significant correlation was identified between candidate ARGs and M2 TAMs. These results revealed that the prognostic signature and ARGclusters constructed by ARGs can distinguish the difference of tumor immune cells in COAD. Compared with the hot tumor, the cold tumor means that there are fewer immune cells infiltrating in the tumor, which means that the response to immunotherapy is weaker (46). Our results indicated that patients in ARGclusterA and low-risk group belonged to the cold tumor subtype. The proportion of immune cells and ESTIMATE score in the ARGclusterA and low-risk group were significantly lower, which consistent with the difintion of “immune-desert” phenotype (47). The above results revealed that immune monitoring function of patients in ARGclusterA and low-risk group was weakened, which was conducive to immune escape, and the effect of immunotherapy was poor.

Compared with left-sided CRC (LCRC), right-sided CRC (RCRC) is usually associated with poor prognosis, and also presents more advanced N stage, larger tumor size, poorly differentiated tumors, as well as higher probability of lymphatic vascular invasion (48). In addition, RCRC also presented higher hypermethylation and higher microsatellite instability (MSI) frequency than LRCR (26, 49). Higher risk score was associated with worse survival rate and higher MSI-high proportion, which was consistent with phenotypic characteristics of RCRC. Moreover, our results revealed that angiogenesis-related signature had a strong ability to discriminate LCRC and RCRC.

Previous studies on microsatellite stable CRC noted that patients with high TMB have longer median survival time (50). In most cancers, the higher the TMB level, the longer the OS of patients after immunotherapy (51, 52). About 12% to 15% of all CRC patients are MSI-H/dMMR (53).It was concluded that in early stages of CRC, MSI-H/dMMR CRC patients have a good prognosis, but in patients with mCRC, this status is associated with a poor prognosis (54). Immunotherapy in advanced CRC patients with MSI-H/dMMR has a high efficiency and can improve the OS of patients (55). We found a higher percentage of MSI-H in the high-risk group, while they had a higher TMB, which suggest that they are more sensitive to immunotherapy.

The development of resistance to chemotherapy in colorectal cancer is often a problem for physicians and patients (56). COAD patients with higher ARG_score were more sensitive to Cisplatin, Docetaxel, Gemcitabine, Paclitaxel, Obatoclax.mecylate, and Vinblastine, which means that the effect of chemotherapeutic drugs was better in the high-risk groups. Our findings may provide more evidence for the follow-up study of ARGs and tumor resistance, which may help to reduce drug resistance and improve clinical outcomes.

This study has shortcomings. All conclusions of the article were derived from the processing of data from public databases and retrospective analysis, and prospective clinical studies are lacking to validate the results. In addition, our analysis lacks *in vivo* and *in vitro* experiments to corroborate accuracy of this model in depth.

## Conclusion

In summary, we constructed a risk score model for assessing the prognosis, immune infiltration, and drug sensitivity of COAD patients. The results of this study facilitate individualized assessment of patient prognosis and drug therapy in clinical.

## Data availability statement

The datasets presented in this study can be found in online repositories. The names of the repository/repositories and accession number(s) can be found in the article/**Supplementary Material**.

## Author contributions

ZGS and FW conceived the study and participated in the study design and manuscript writing. GLC, JHL and MLW conducted the bioinformatics analysis. KYS and CW revised the manuscript. All authors read and approved the final manuscript.

## Acknowledgments

We would like to extend our gratitude to the researchers and study patients for their contributions.

## Conflict of interest

The authors declare that the research was conducted in the absence of any commercial or financial relationships that could be construed as a potential conflict of interest.

## Publisher’s note

All claims expressed in this article are solely those of the authors and do not necessarily represent those of their affiliated organizations, or those of the publisher, the editors and the reviewers. Any product that may be evaluated in this article, or claim that may be made by its manufacturer, is not guaranteed or endorsed by the publisher.

## Supplementary material

Supplementary Figure 1 Kaplan-Meier survival analysis of different angiogenesis-related genes in COAD. The red and blue line represent patients with higher expression and lower expression of ARGs respectively.

Supplementary Figure 2 1587 differentially expressed gene (DEGs) between two ARGclusters were showed in Venn diagram.

Supplementary Figure 3 Prognostic value of ARGs in training set. **(A, B)** Multivariate Cox regression *via* LASSO is presented, and eight candidate ARGs were selected in training cohort.

Supplementary Figure 4 The expression level of 8 angiogenesis-related genes. **(A–F)** Compared with normal colon epithelial cells, SEMA4C, and ASNS expression were significantly increased in three CRC cell lines. PIM1, JAGN1 and RPS24 expression were significantly increased in Caco-2 and HT29 cells, but not in HCT-116 cells. TRIB2 expression was significantly increased in HT-29 and HCT-116 cells, but not in Caco- 2 cells.

Supplementary Figure 5 Stratification analysis of the ARG_score in COAD. **(A–D)** In terms of survival status and tumor stage, the proportion of deaths and stage III-IV tumors was significantly higher in the high-risk group than that in the low-risk group; in terms of age and gender, there were no significant differences between the two risk groups. **(E–G)** Kaplan-Meier curves depicted the survival difference between lower and higher ARG_score in the stratified analysis of COAD patients. Survival expectations were lower in the high-risk group than in the low-risk group, regardless of age, gender, or tumor stage.

Supplementary Figure 6 The correlation of risk score and different sided colorectal cancer (CRC). **(A)** The proportion of right-sided CRC (RCRC) and left-sided CRC (LCRC) patients in the different risk group. **(B)** The risk score of patients with RCRC and LRCR, higher risk score was found in RCRC.

Supplementary Figure 7 The difference of immune cells and immune functions between two risk groups.
