# Supplementary material for: Crosstalk of angiogenesis-related subtypes, establishment of a prognostic signature and immune infiltration characteristics in colorectal adenocarcinoma
Source: Front Immunol. 2022 Nov 24;13:1049485. doi: 10.3389/fimmu.2022.1049485 (PMC9731117; doi:10.3389/fimmu.2022.1049485)
Supplement: Supplementary file 1 [file Table_1.docx]

| Covariates | Type | Total | Test | Train | Pvalue |
| --- | --- | --- | --- | --- | --- |
| Age | <=65 | 403(40.87%) | 172(43.77%) | 231(38.95%) | 0.1503 |
|  | >65 | 583(59.13%) | 221(56.23%) | 362(61.05%) |  |
| Gender | FEMALE | 448(45.44%) | 190(48.35%) | 258(43.51%) | 0.1531 |
|  | MALE | 538(54.56%) | 203(51.65%) | 335(56.49%) |  |
| Stage | Stage I | 109(11.05%) | 39(9.92%) | 70(11.8%) | 0.7523 |
|  | Stage II | 425(43.1%) | 168(42.75%) | 257(43.34%) |  |
|  | Stage III | 325(32.96%) | 133(33.84%) | 192(32.38%) |  |
|  | Stage IV | 116(11.76%) | 49(12.47%) | 67(11.3%) |  |
|  | unknow | 11(1.12%) | 4(1.02%) | 7(1.18%) |  |
| T | T1 | 21(2.13%) | 11(2.8%) | 10(1.69%) | 0.4208 |
|  | T2 | 122(12.37%) | 43(10.94%) | 79(13.32%) |  |
|  | T3 | 656(66.53%) | 262(66.67%) | 394(66.44%) |  |
|  | T4 | 166(16.84%) | 70(17.81%) | 96(16.19%) |  |
|  | unknow | 21(2.13%) | 7(1.78%) | 14(2.36%) |  |
| M | M0 | 798(80.93%) | 318(80.92%) | 480(80.94%) | 0.7508 |
|  | M1 | 117(11.87%) | 49(12.47%) | 68(11.47%) |  |
|  | unknow | 71(7.2%) | 26(6.62%) | 45(7.59%) |  |
| N | N0 | 550(55.78%) | 213(54.2%) | 337(56.83%) | 0.776 |
|  | N1 | 232(23.53%) | 98(24.94%) | 134(22.6%) |  |
|  | N2 | 172(17.44%) | 69(17.56%) | 103(17.37%) |  |
|  | N3 | 6(0.61%) | 3(0.76%) | 3(0.51%) |  |
|  | unknow | 26(2.64%) | 10(2.54%) | 16(2.7%) |  |

**Table S1 Distribution of patients into training cohort, testing cohort and total cohort.**

**Table S2 Primer sequence of genes in qRT-PCR.**

|  | Forward sequence | Reverse sequence |
| --- | --- | --- |
| SEMA4C | TTGTGCCGCGTAAGACAGTG | CCGTCAGCGTCAGTGTCAG |
| PIM1 | GGCTCGGTCTACTCAGGCA | GGAAATCCGGTCCTTCTCCAC |
| JAGN1 | TCGCCATGCACTACCAGATG | AAATGCTCAGCAAATACGGGT |
| TRIB2 | ATGAACATACACAGGTCTACCCC | GGGCTGAAACTCTGGCTGG |
| ASNS | GGAAGACAGCCCCGATTTACT | AGCACGAACTGTTGTAATGTCA |
| RPS24 | ATGAACGACACCGTAACTATCCG | CCGAATTTCTGTCTTAGGCACTG |
| β-actin | CATGTACGTTGCTATCCAGGC | CTCCTTAATGTCACGCACGAT |
