# Supplementary figures and images for: Crosstalk of angiogenesis-related subtypes, establishment of a prognostic signature and immune infiltration characteristics in colorectal adenocarcinoma
Source: Front Immunol. 2022 Nov 24;13:1049485. doi: 10.3389/fimmu.2022.1049485 (PMC9731117; doi:10.3389/fimmu.2022.1049485)

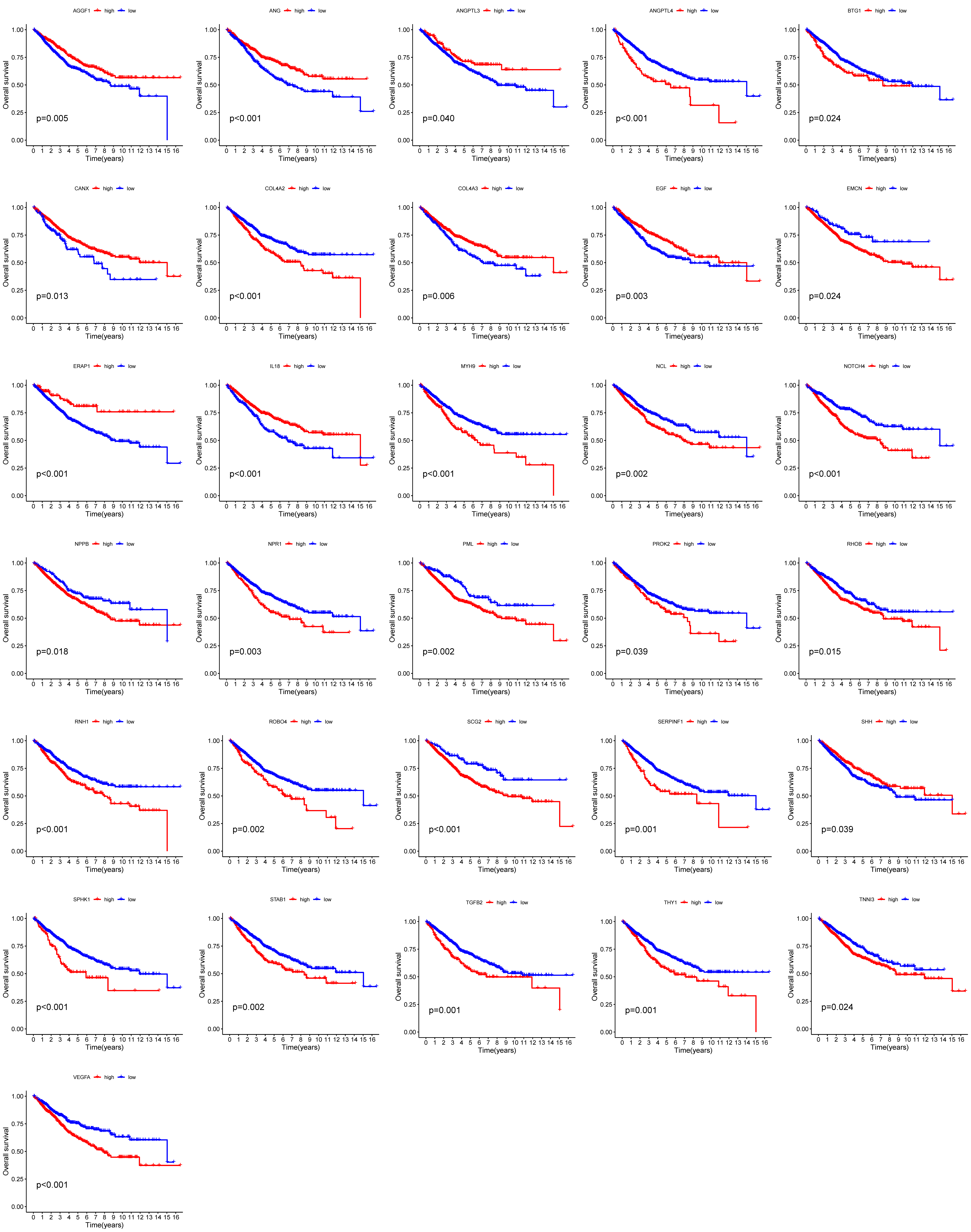

Supplement: Supplementary Figure 1 — Kaplan-Meier survival analysis of different angiogenesis-related genes in COAD. The red and blue line represent patients with higher expression and lower expression of ARGs respectively. [file Image_1.tif]

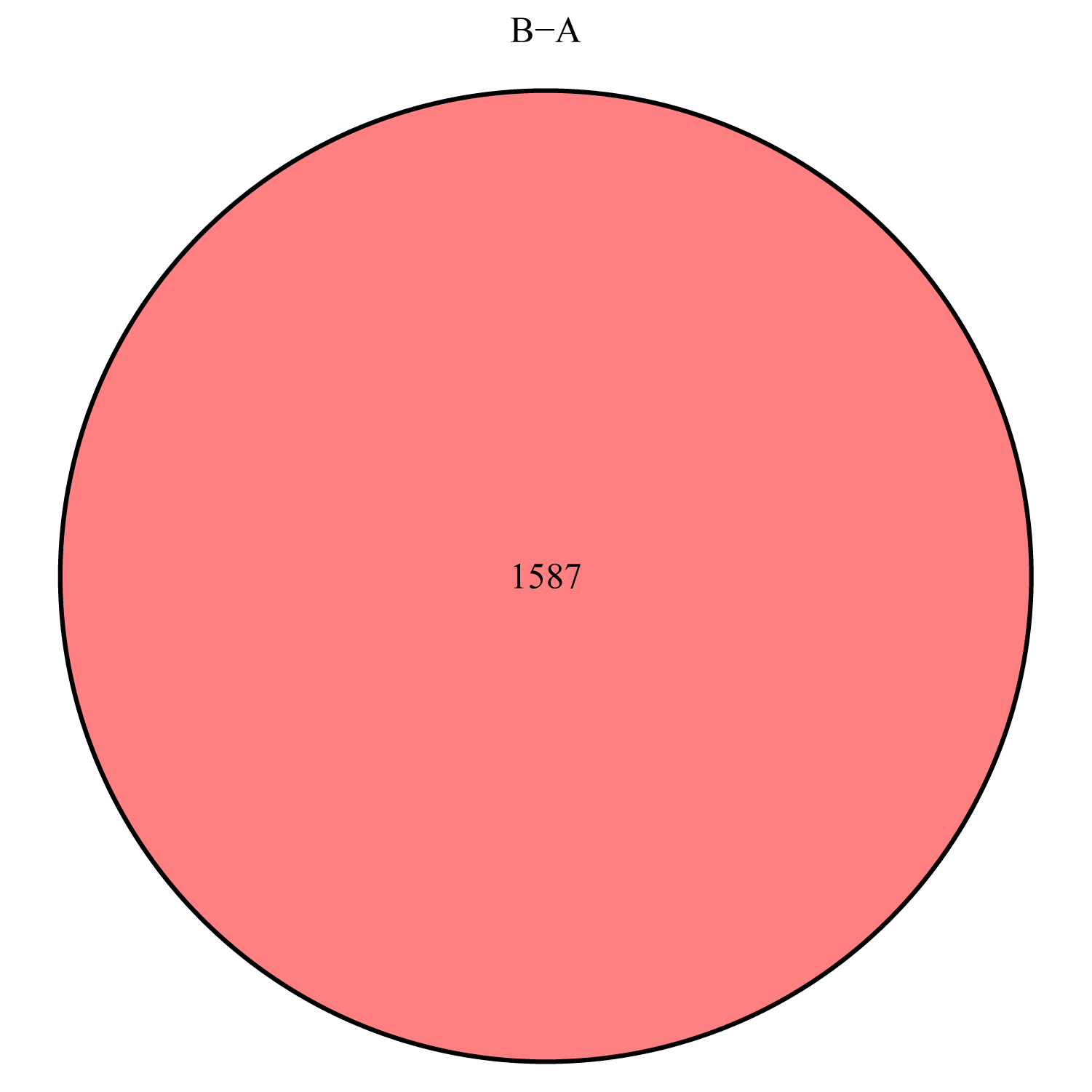

Supplement: Supplementary Figure 2 — 1587 differentially expressed gene (DEGs) between two ARGclusters were showed in Venn diagram. [file Image_2.tif]

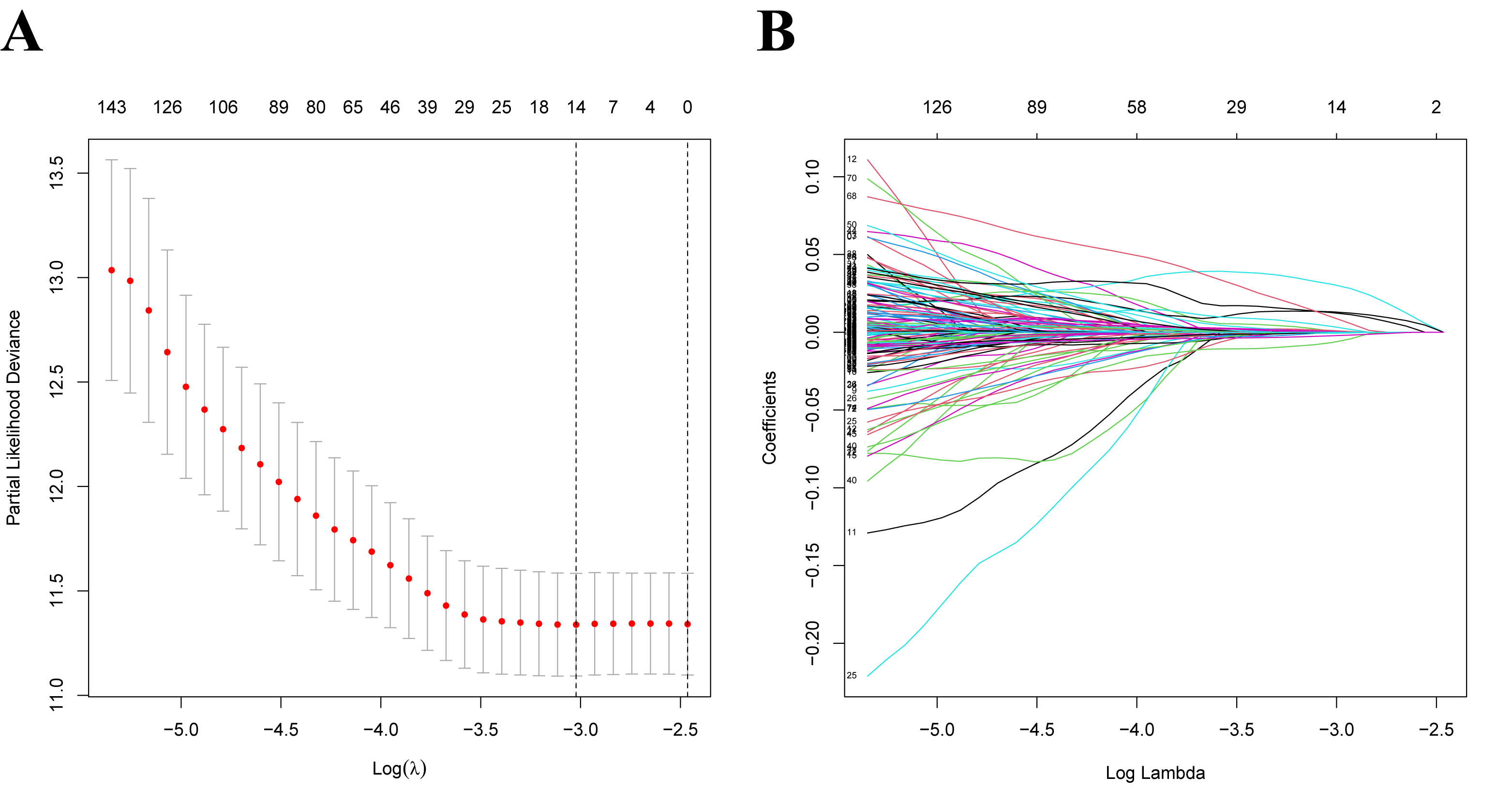

Supplement: Supplementary Figure 3 — Prognostic value of ARGs in training set. (A, B) Multivariate Cox regression via LASSO is presented, and eight candidate ARGs were selected in training cohort. [file Image_3.tif]

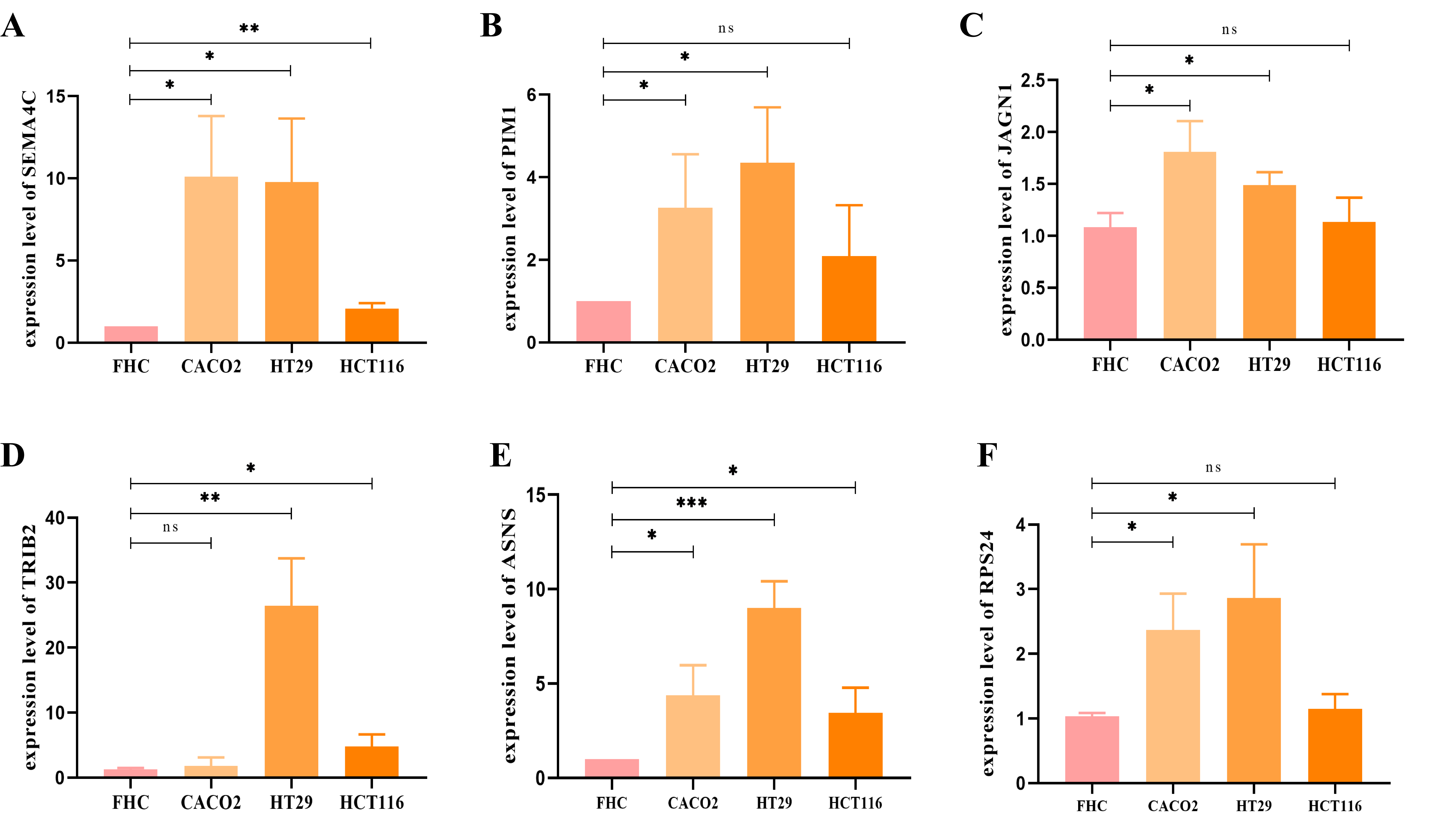

Supplement: Supplementary Figure 4 — The expression level of 8 angiogenesis-related genes. (A–F) Compared with normal colon epithelial cells, SEMA4C, and ASNS expression were significantly increased in three CRC cell lines. PIM1, JAGN1 and RPS24 expression were significantly increased in Caco-2 and HT29 cells, but not in HCT-116 cells. TRIB2 expression was significantly increased in HT-29 and HCT-116 cells, but not in Caco- 2 cells. [file Image_4.tif]

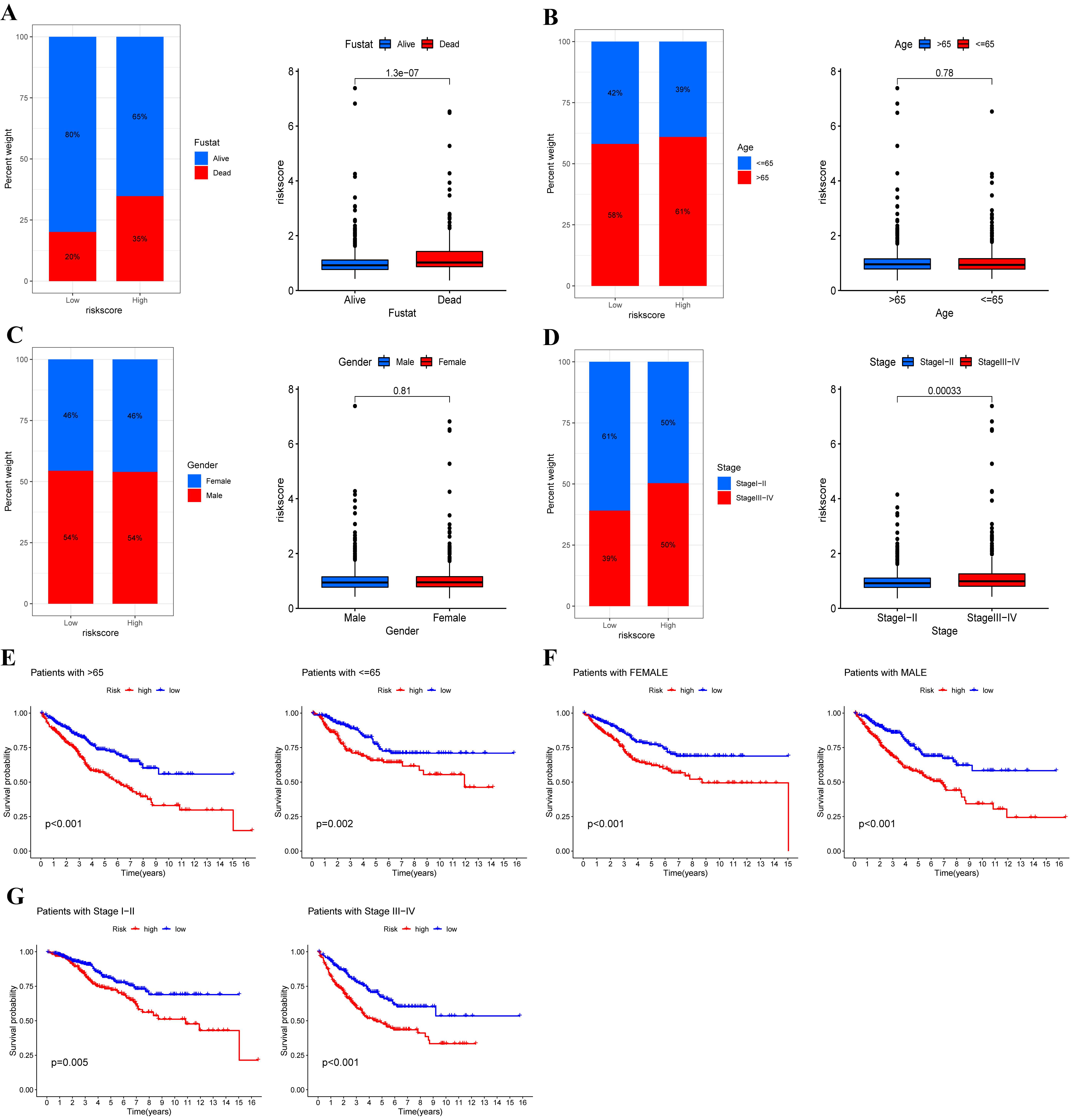

Supplement: Supplementary Figure 5 — Stratification analysis of the ARG_score in COAD. (A–D) In terms of survival status and tumor stage, the proportion of deaths and stage III-IV tumors was significantly higher in the high-risk group than that in the low-risk group; in terms of age and gender, there were no significant differences between the two risk groups. (E–G) Kaplan-Meier curves depicted the survival difference between lower and higher ARG_score in the stratified analysis of COAD patients. Survival expectations were lower in the high-risk group than in the low-risk group, regardless of age, gender, or tumor stage. [file Image_5.tif]

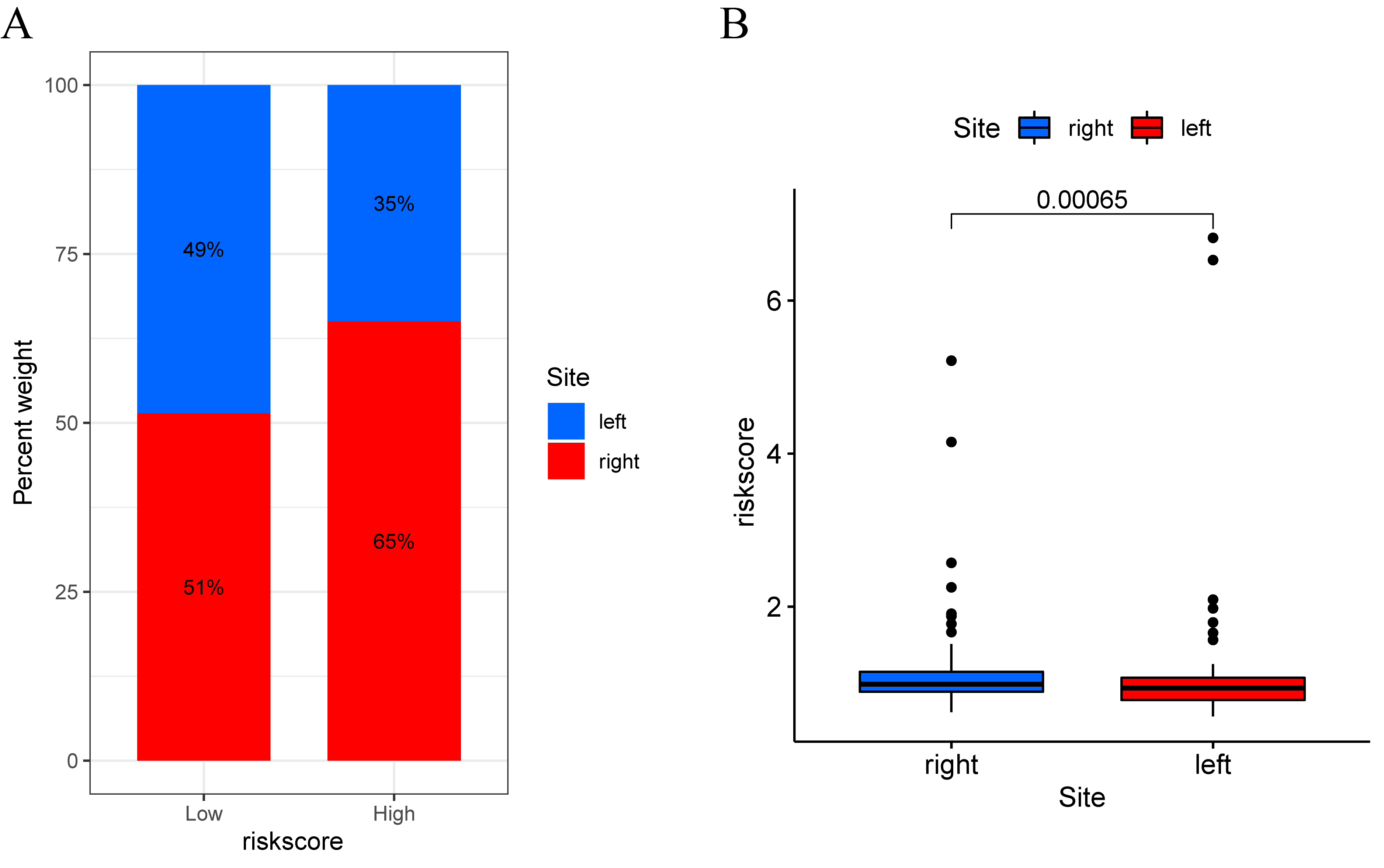

Supplement: Supplementary Figure 6 — The correlation of risk score and different sided colorectal cancer (CRC). (A) The proportion of right-sided CRC (RCRC) and left-sided CRC (LCRC) patients in the different risk group. (B) The risk score of patients with RCRC and LRCR, higher risk score was found in RCRC. [file Image_6.tif]

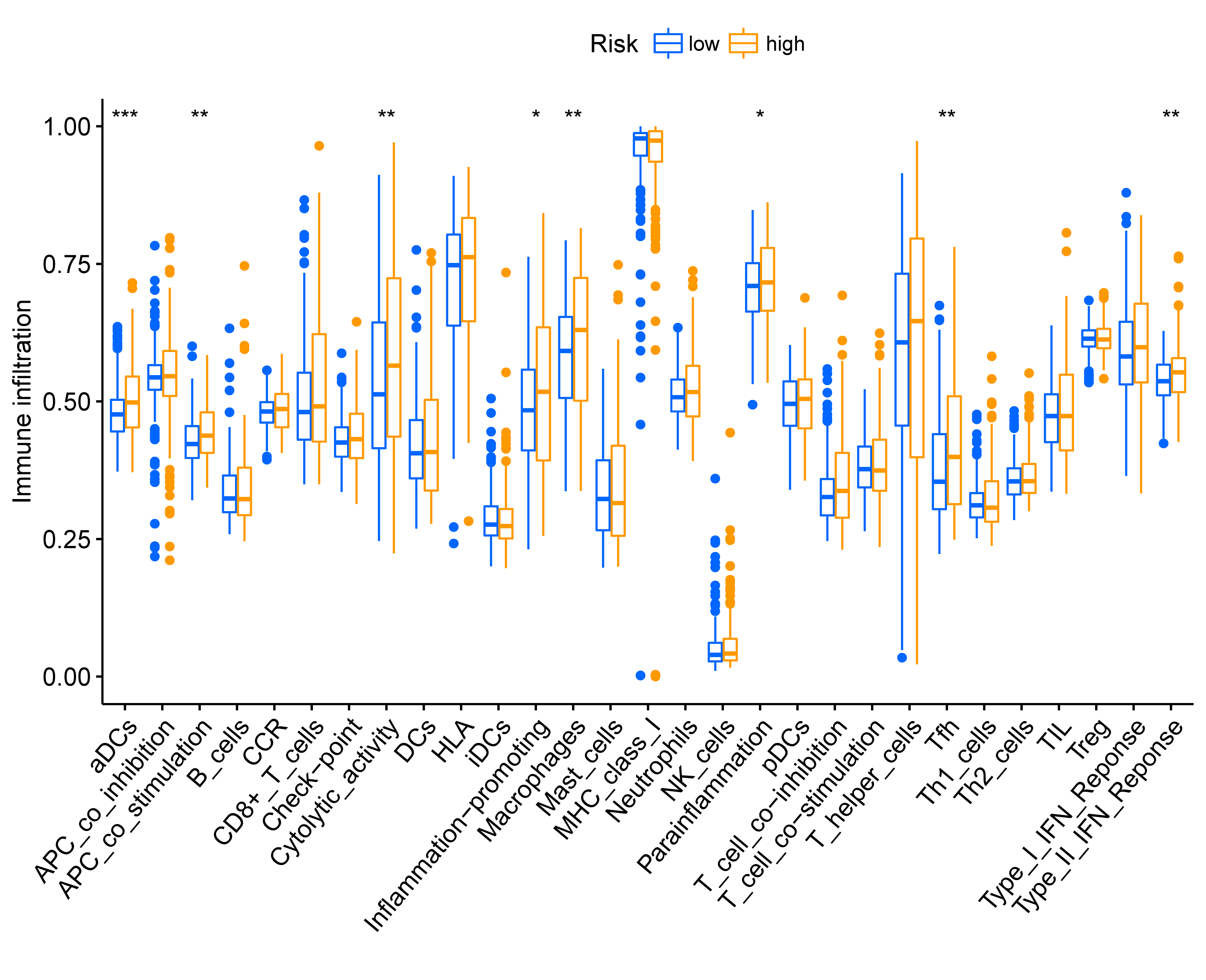

Supplement: Supplementary Figure 7 — The difference of immune cells and immune functions between two risk groups. [file Image_7.tif]
